# Online Dating and Mental Health among Young Sexual Minority Black Men: Is Ethnic Identity Protective in the Face of Sexual Racism?

**DOI:** 10.3390/ijerph192114263

**Published:** 2022-11-01

**Authors:** Ryan M. Wade, Matthew M. Pear

**Affiliations:** School of Social Work, University of Illinois Urbana-Champaign, Champaign, IL 61820, USA

**Keywords:** sexual racism, ethnic identity, gay/bisexual men, mobile apps, mental health

## Abstract

Racialized Sexual Discrimination (RSD), also known as ‘sexual racism,’ is pervasive within online dating venues. RSD is associated with poor mental health outcomes among young sexual minority Black men (YSMBM), and there is limited research on factors that may mitigate this association. Ethnic identity has been identified as a potential protective factor for racial/ethnic minorities who encounter racialized stressors, though some evidence suggests that ethnic identity may also intensify the negative effects of racial discrimination. Using data from a cross-sectional web-survey of YSMBM (*n* = 690), a series of linear regression models were estimated to examine the moderating effect of ethnic identity search and ethnic identity commitment on the relationship between RSD and depressive symptoms/feeling of self-worth. Results indicated that having moderate-to-high scores on commitment attenuated the association between being physically objectified by White men and higher depressive symptoms. However, having high scores on commitment intensified the association between being rejected by Black men and lower feelings of self-worth. Stronger identity commitment may be protective against objectification from White men, though it may also exacerbate negative outcomes related to in-group discrimination. These findings may have important implications for the development of individual and group-level interventions addressing ethnic identity among YSMBM.

## 1. Introduction

Online intimate partner-seeking is now widespread among Americans, especially among sexual minority adolescents and young adults. Young sexual minority populations use dating apps and websites at higher rates than their older and heterosexual counterparts, and use of such platforms has been increasing over the past decade [1,2]. Sexual minority men of color face frequent discrimination on these dating apps and websites, a phenomenon referred to as Racialized Sexual Discrimination (RSD) [3,4]. RSD is a multidimensional construct that encompasses a variety of racially mediated discriminatory experiences—such as overt and covert rejection on the basis of race, and the positioning of White men as superior or more desirable than other racial/ethnic minority groups—all of which have been well-documented in the literature [4,5,6,7,8,9]. Users may express their sexual “preferences” based on race directly on their profiles (e.g., writing “White men only;’ “not into Black guys, etc.); users may also systematically ignore messages from men of color, or explicitly reject men of color on the basis of their racial/ethnic minority background. Erotic objectification is also commonly reported on these platforms and is another key dimension of RSD. Patterns of objectification are largely driven by racial stereotypes that are deeply ingrained in American and many other Western cultures. For Black men, these stereotypes often cast them as having large penises, as well as being physically imposing, dominant, and aggressive [10,11,12,13,14,15].

The phenomenon of RSD is situated within a broader literature of sexual racism. Sexual racism has been described as the systemic ways in which individuals and society establish a racial hierarchy of desire—wherein White identity/Eurocentric features are considered ideal, interracial intimacy is less socially accepted, and the erotic capital of racial/ethnic minorities are altogether diminished, or wholly afforded through racial fetishization [16,17,18]. As a contemporary online phenomenon, RSD has been receiving growing attention in the social science and health literature [19,20,21,22,23]. Recently, researchers have demonstrated links between RSD and negative health outcomes, such as anxiety and psychological distress—as well as lower self-esteem and well-being among sexual minority men of color [24,25,26,27,28,29]. In a series of recent studies, we developed the first psychometrically evaluated scale of RSD and examined the association between RSD and psychological well-being among young sexual minority Black men (YSMBM) [30,31]. We found that encountering same-race rejection and White superiority when seeking intimate partners online was associated with higher depressive symptoms among YSMBM. We also found that encountering objectification from White men was associated with both higher depressive symptoms and lower feelings of self-worth among the study sample. Altogether, the emerging literature on RSD has provided evidence that this unique and understudied phenomenon may negatively impact the health of sexual minority men of color.

### 1.1. Theoretical Underpinnings of Identity, Discrimination, and Health

Much of our work on RSD has been grounded in Meyer’s Minority Stress Theory (MST), which provides an apt framework for understanding how RSD contributes to adverse health outcomes among sexual minority populations [32]. MST explicates the mechanisms through which distal and proximal identity-related stressors lead to poor mental health outcomes among marginalized populations, drawing special attention to the role of identity in the experience of stress—such as race- or sexuality-based discrimination. Meyer posits that characteristics of an individual’s minoritized identity—such as the extent to which an individual identifies with their minority status (salience) and their evaluation of that identity (valence)—modulates the experience of identity-related stress and subsequent health outcomes. However, there is limited research focusing specifically on ethnic identity among YSMBM, and how ethnic identity operates in the context of race-related stress within this population.

There are a number of theories addressing ethnic identity that may complement MST and provide important nuance in investigating RSD among sexual minority men of color. Building upon Erickson’s and Marcia’s seminal work on adolescent identity development [33,34,35], Phinney pioneered one of the most well recognized frameworks of ethnic identity [36,37], culminating in the creation of the widely used Multi-Ethnic Identity Measure (MEIM). In subsequent psychometric and construct validity work, Phinney established two key components of ethnic identity: identity search/exploration and identity commitment. Identity search/exploration refers to the effort that individuals expend in learning about, and making meaning of, their ethnic group membership. Identity commitment refers to individuals’ sense of belonging to their ethnic group, as well as how they appraise the relative value of belonging to their ethnic group.

Both Phinney and other scholars have suggested that higher levels of identity commitment may confer protective benefits and partially mitigate the effects of racial discrimination [36,38,39,40]. Having a robust and assured sense of self, as well as finding strength and solidarity through one’s group membership, is thought to deflect threats to an individual’s self-concept—thereby preventing identity-based attacks (i.e., racial discrimination) from negatively impacting psychosocial functioning [36,41,42]. Identity search/exploration, however, has been hypothesized to operate differently in the context of discrimination among some scholars. While not strictly negatively valenced, identity search/exploration is thought to represent a lack of clarity or full integration of one’s sense of ethnic identity (hence the need to explore). This uncertainty may make an individual more vulnerable to racial discrimination or prejudice, thereby acting as an exacerbating characteristic in the context of identity-related stress [39,40,43]. Though these two aspects of ethnic identity are conceptually distinct, researchers have mostly examined identity search and commitment in tandem (i.e., a composite measure is most often used), and the researchers who have disaggregated these two constructs have largely only focused on Latino and Asian men in their study samples [39,44,45,46]. Moreover, there is considerably less research examining ethnic identity among sexual minority populations in general, and among YSMBM in particular [47]. The ways in which ethnic identity functions as a protective or exacerbating characteristic in the context of race-related stress is ultimately an ongoing empirical question, especially among populations that have historically been underexamined—and in the context of understudied racialized stressors, such as RSD.

### 1.2. Empirical Findings on the Moderating Role of Ethnic Identity

There is an extensive body of empirical literature on ethnic identity in general, with most researchers reporting that stronger ethnic identity is associated with positive psychosocial outcomes among Black Americans and other racial/ethnic minority groups [47,48,49,50,51,52]. However, researchers have reported far more nuanced findings with respect to how identity search and commitment operates in the context of race-related stress. Among a sample of Latino adults, Torres et al. reported that ethnic identity exploration exacerbated the association between public, work-related, and academic-related discrimination and psychological distress [46]. In contrast, ethnic identity commitment attenuated the association between covert discrimination and negative mental health outcomes. Torres and Ong reported near identical findings in a comparable study, in which they investigated the association between daily discrimination and next-day depression among Latino adults [39]. Identity exploration again exacerbated the association between discrimination and depression, whereas identity commitment acted as a buffer. These findings coincide with theories of ethnic identity that position commitment as a protective characteristic and exploration as an exacerbating characteristic in the context of race-related stress.

In a study of online discrimination and well-being, Tynes et al. reported that that ethnic identity buffered the association between online discrimination and anxiety symptoms among Black adolescents, but did not significantly alter the association between online discrimination and depressive symptoms [53]. Romero and Roberts similarly reported that high levels of identity commitment buffered the effects of discrimination on Latino adolescents’ self-esteem, and they also found that low levels of identity commitment exacerbated this association [54]. Other researchers have also found that ethnic identity serves as a protective factor in the context of racial discrimination and adverse psychosocial health outcomes, though these researchers used ethnic identity as a composite measure [51,55]. In such cases, it is difficult to determine whether these associations are driven by both commitment and exploration, or one over the other. In all cases, however, researchers have not expressly focused on the experiences of sexual minority populations of color. This points to a significant gap in our understanding given the added complexity of intersectional identity, and the elevated risk associated with holding multiple oppressed identities [56,57].

### 1.3. Does Ethnic Identity Buffer or Exacerbate the Impact of RSD?

There are two key benefits of examining ethnic identity as a moderator between RSD and health. First, the theoretical and empirical literature has indicated that not all characteristics of ethnic identity are uniformly protective—and in some instances, certain aspects of ethnic identity may actually worsen the negative effects of discrimination [39,40,43,58,59]. Understanding which aspects of identity are protective—and which are not—may be crucial in developing culturally tailored interventions that address discrimination and psychological well-being. Second, ethnic identity has been minimally investigated among YSMBM in general, and in the context of RSD specifically. The very personal and often vulnerable nature of intimate partner-seeking makes RSD a unique racialized stressor. That RSD is defined as an online-specific phenomenon also makes it unique—given that discrimination is typically more brazen, prejudices more overt, and rejection more frequent–in high density, quasi-anonymous settings such as online venues [60,61,62,63,64]. The novel RSD scale used in this study captures a broad scope of these experiences and distinguishes between expressions of discrimination to a greater degree than most measures of discrimination [30]. Its distinction between race of perpetrator (same-race and White perpetration of RSD) is especially useful, as there is a noteworthy deficit of research that accounts for differences between in-group and out-group discrimination in the context of RSD. Examination of same-race perpetration of RSD is rare in general, though we have found that YSMBM report complex attitudes toward same-race perpetration of RSD in previous work [65].

Given the importance of ethnic identity in the context of race-related stress, the current study aims to examine the ways in which ethnic identity might serve to modify the relationship between RSD and psychological well-being among YSMBM. In accordance with prior theoretical and empirical work, we hypothesized that (**Hypothesis 1; H1**) higher scores on RSD would be associated with poorer psychological well-being among the study sample; (**Hypothesis 2; H2**) higher scores on ethnic identity search would exacerbate the association between RSD and well-being; and (**Hypothesis 3; H3**) higher scores on ethnic identity commitment would attenuate the association between RSD and well-being.

## 2. Materials and Methods

### 2.1. Participants

Eligibility Criteria. In order to be eligible for the study, participants had to meet the following criteria: (1) identify as a man; (2) be assigned male sex at birth; (3) identify primarily as Black, African American, or with any other racial/ethnic identity across the African diaspora (e.g., Afro-Caribbean, African, etc.); (4) be between the ages of 18 and 29 inclusive; (5) identify as gay, bisexual, queer, same-gender-loving, or another non-heterosexual identity, or report having had any sexual contact with a man in the last 3 months; (6) report having used a website or mobile app to find male partners for sexual activity in the last 3 months; and (7) reside in the United States.

### 2.2. Recruitment

A non-probability convenience sample of YSMBM were recruited using best practices for online survey sampling [66,67] between July 2017 and January 2018. Participants were primarily recruited through Facebook and Scruff, a mobile app for gay and bisexual men to meet one another for sex or dating. Prospective participants viewed advertisements for the study and clicked on a link embedded in the advertisement that directed them to the study webpage. Advertisements on Facebook were only made viewable to men in the targeted age range who lived in the United States. Facebook ads were further tailored to target individuals who (1) indicated that they were “interested in” men, or who omitted information on the gender in which they were interested; (2) indicated interest in various LGBTQ-related pages on Facebook; (3) matched Facebook’s behavior algorithms for U.S. African American Multicultural Affinity; or (4) indicated interest in various pages related to popular Black culture.

### 2.3. Procedure

Prospective participants were directed to a survey hosted on Qualtrics upon clicking on the study advertisement. Participants were presented with a set of screening questions to determine their eligibility. Those who met the eligibility criteria were directed to a consent page, which contained detailed study information (i.e., purpose of the research, description of participant involvement, risk/discomforts; benefits; confidentiality, etc.). Those consenting to participate proceeded to the full survey which lasted 30 to 45 min. Participants were not compensated for taking the survey. While completing the survey, participants were permitted to save their answers and return to the survey at a later time if they were not able to complete it in a single sitting. Study data were kept in an encrypted and firewall-protected server, and the Institutional Review Board at the University of Michigan all study procedures.

### 2.4. Measures

**Outcome Variables**. The two dependent variables used in this study include Depressive Symptoms and Feelings of Self-Worth.

*Depressive Symptoms.* We measured depressive symptoms using the Center for Epidemiologic Studies Depression scale [68,69]. Participants were presented with a series of statements (e.g., ‘I thought my life had been a failure’) and were asked to indicate how often they have experienced each one. Each item was measured on a 4-point Likert scale ranging from 0 (Rarely or none of the time) to 3 (Most or all of the time). The mean of 20 items was computed to generate an overall CES-D score. Four items on the scale were reverse coded so that all responses were in directional alignment; higher scores indicate higher self-reported levels of depressive symptoms in the past week. The Cronbach’s alpha value for depressive symptoms demonstrated excellent reliability (α = 0.920).

*Feelings of Self-Worth.* We measured self-worth using the Feelings of Self-Worth Measure [70]. Participants were asked to indicate the degree to which they agree with a series of statements (e.g., ‘I feel good about myself right now’). Each item was measured on a 9-point Likert scale ranging from 0 (Not at all) to 8 (Extremely). The mean of 14 items was computed to generate a self-worth score. Seven items on the scale were reverse coded so that all responses were in directional alignment; higher scores indicate higher self-reported feelings of self-worth. The Cronbach’s alpha value for feelings of self-worth demonstrated excellent reliability (α = 0.950).

**Moderator Variables.** We measured two dimensions of ethnic identity using the Multiethnic Identity Measure (MEIM) [37]. Participants were asked to indicate the degree to which they agreed with a series of statements (e.g., ‘In order to learn more about my ethnic background, I have often talked to other people about my ethnic group;’ ‘I feel a strong attachment towards my own ethnic group’). Each item was measured on a 4-point Likert scale ranging from 1 (Strongly disagree) to 4 (Strongly agree). For the first subscale, the mean of 5 items was computed to generate an ethnic identity search (EIS) score. For the second subscale, the mean of 7 items was computed to generate an ethnic identity commitment (EIC) score. Higher scores indicate higher self-reported ethnic identity for each subscale. The Cronbach’s alpha value for EIS (α = 0.780) and EIC (α = 0.907) demonstrated acceptable to excellent reliability.

**Covariates.** The covariates in this study include self-perceived sexual attractiveness, perceived rejection, mobile app/website use for partner seeking, and four sociodemographic variables (age, relationship status, HIV status, and educational attainment). Sexual orientation is reported for descriptive purposes only.

*Self-Perceived Sexual Attractiveness.* We measured Self-Perceived Sexual Attractiveness (SPSA) using the SPSA scale [71]. Participants were asked to indicate the degree to which they agreed with a series of statements (e.g., ‘I believe I can elicit sexual desire in other people’). Each item was measured on a 7-point Likert scale ranging from 1 (Strongly disagree) to 7 (Strongly agree). The mean of 6 items was computed to generate an SPSA score. Higher scores indicate higher self-reported levels of SPSA. The Cronbach’s alpha value for SPSA demonstrated excellent reliability (α = 0.951).

*Perceived Rejection*. We measured sensitivity to rejection using the Perceived Rejection Scale [72]. Participants were asked to indicate the degree to which a series of statements was true at the immediate moment (e.g., ‘I am accepted by others’). Each item was measured on a 5-point Likert scale ranging from 0 (Not at all) to 4 (Extremely). The mean of 4 items was computed to generate a perceived rejection score. Two items on the scale were reverse coded so that all responses were in directional alignment; higher scores indicate higher self-reported levels of perceived rejection. The Cronbach’s alpha value for perceived rejection demonstrated acceptable reliability (α = 0.761).

*Sociodemographics.* Participants were instructed to enter their numerical age. Participants could indicate a response of ‘yes’ or ‘no’ when asked if they were single and when asked if they have ever tested positive for HIV. Frequency of mobile app/website use to find partners was measured using a 6-point Likert scale containing the following values: 1 = ‘Once a month or less;’ 2 = ‘2–3 times a month;’ 3 = ‘About once a week;’ 4 = ‘2–6 times a week;’ 5 = ‘About once a day;’ 6 = ‘More than once a day.’ Educational attainment was measured using a 5-point Likert scale containing the following values: 1 = ‘Less than high school;’ 2 = ‘High school graduate;’ 3 = ‘Some college;’ 4 = ‘College graduate;’ 5 = ‘Post College.’ Finally, participants could select one of 11 sexual orientation categories (e.g., Gay, Bisexual, Queer, etc.)

**Independent Variables.** Data were collected on participants’ self-reported experiences of sexual racism using the Racialized Sexual Discrimination Scale (RSDS) [30]. Each experience on the scale has two corresponding items: one that captures the effect (i.e., to what degree the experience has a negative effect on the participant) and the frequency (i.e., how often a participant encounters the experience). Experiences described on the scale could occur in one of two contexts: partner browsing (i.e., viewing user profiles on mobile apps/websites) and partner negotiation (i.e., written communication between users on mobile apps/websites). Items within the partner browsing context were measured on a 5-point Likert scale ranging from 0 (Strongly disagree) to 4 (Strongly agree). Items within the partner negotiation context were measured on a 6-point Likert scale ranging from 0 (I have not contacted this group) to 5 (Strongly agree).

The effect and frequency scores for each item within the partner browsing context were multiplied to develop an impact score, ranging from 0 to 16. This impact score was divided by 16 and multiplied by 100 to result in a final impact score for each partner browsing item, ranging from 0 to 100. Likewise, the effect and frequency scores for each item within the partner negotiation context were multiplied to develop an impact score, ranging from 0 to 25. For ease of interpretation, this impact score was divided by 25 and multiplied by 100 to result in a final impact score for each partner negotiation item, ranging from 0 to 100. Subsequently, all partner browsing and partner negotiation item impact scores ranged from 0 to 100, where higher scores indicate higher overall impact of RSD.

*White Superiority, White Physical Objectification, and Same-Race Rejection.* The White superiority subscale score was computed using the mean of 8 impact items (e.g., ‘When White people clearly state that they want to meet other White people, I have a negative reaction’). The White physical objectification subscale score was computed using the mean of 2 impact items (e.g., ‘How often do White people express a desire for a specific physical trait related to your race/ethnicity?’). The same-race rejection subscale score was computed using the mean of 2 impact items (e.g., ‘How often are your messages ignored by people of your own race/ethnicity?). The Cronbach’s alpha value for White superiority (α = 0.833), White physical objectification (α = 0.857), and same-race rejection (α = 0.851) demonstrated strong reliability.

### 2.5. Data Analytic Strategy

A total of 2188 eligible and consenting participants were recruited for the study. Participants with missing data were excluded, resulting in a final analytic sample of 690 participants. Descriptive statistics were computed for the study sample, including mean scores, frequency counts, and percentages for demographic characteristics and study variables. Moderation analyses were conducted using the PROCESS Macro in SPSS [73]. The moderating effect of EIS and EIC on the association between five RSD subscales and two indicators of psychological health (depression and self-worth) was examined. Participants’ sociodemographic characteristics (age, education level, HIV-status, relationship status) and relevant behavioral/psychosocial characteristics (frequency of app/website use for partner seeking, sensitivity to rejection, and self-perceived sexual attractiveness) were modeled as covariates. In total, twelve hierarchical linear regression models were estimated (three for depression and EIS, three for depression and EIC; three for self-worth and EIS, and three for self-worth and EIC). Using the PROCESS analysis output, A simple slope graph was plotted to visualize the conditional effects for all significant interactions.

## 3. Results

### 3.1. Sample Description

The median survey completion time was 33.93 min. The mean age of the sample was 24.47 years (*SD* = 3.18), and most study participants (85.9%) were single. The majority of participants identified as gay (71.2%) or bisexual (16.1%), and a little more than one-eighth of the sample (14.5%) reported being HIV-positive. Nearly one-third of the sample (30.3%) had completed a college degree and more than one-eighth of the sample (15.7%) had received a post-graduate education. Slightly more than two-fifths of the sample (42.6%) had received some college education and only one participant had not completed high school. Participants varied in their app usage, with approximately a quarter of participants (26.1%) reporting a minimum of once-a-day usage, and nearly half of participants (45.6%) reporting less than once-a-week usage. Participants reported moderate levels of self-worth (*M* = 5.56) and low-to-moderate depressive symptoms (*M* = 1.05). Participants also reported low-to-moderate perceived rejection (*M* = 1.49) and moderate to high self-perceived sexual attractiveness (*M* = 5.06). Participants reported overall high ethnic identity search (*M* = 3.08) and commitment (*M* = 3.28) scores on the MEIM (see Table 1).

### 3.2. Regression Analyses

#### 3.2.1. White Superiority

Higher scores on White superiority were associated with higher depressive symptoms in both the EIS (*b* = 0.003, *p* < 0.01, 95% CI [0.001, 0.006]) and EIC models (*b* = 0.004, *p* < 0.01, 95% CI [0.001, 0.006]). Higher scores on EIC were associated with lower depressive symptoms (*b* = −0.104, *p* < 0.01, 95% CI [−0.172, −0.037]) and higher feelings of self-worth (*b* = 0.462, *p* < 0.001, 95% CI [0.272, 0.652]). No significant interaction effects were observed between White superiority and ethnic identity. All four White superiority models were significant (*p* < 0.001) and explained 26–36% of the variance in depressive symptoms and self-worth (see Table 2).

#### 3.2.2. Same-Race Rejection

**Figure 1:**
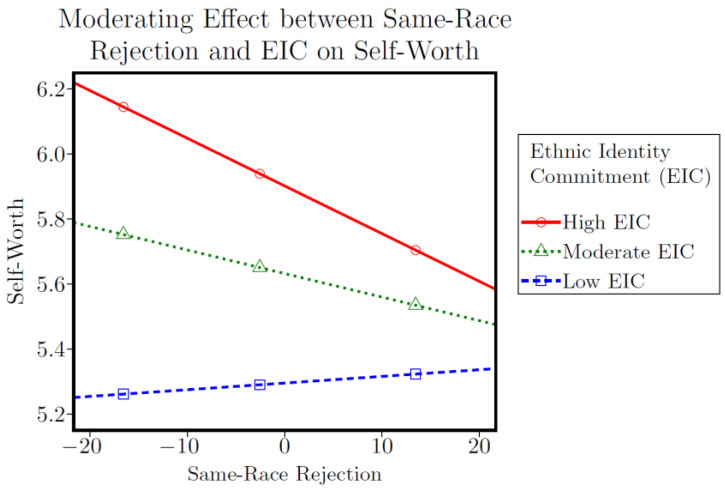
Effect of Same-Race Rejection X EIC on Self-Worth.

Higher scores on same-race rejection were associated with higher depressive symptoms in both the EIS (*b* = 0.005, *p* < 0.001, 95% CI [0.003, 0.008]) and EIC models (*b* = 0.006, *p* < 0.01, 95% CI [0.003, 0.008]). Higher scores on EIC were associated with lower depressive symptoms (*b* = −0.106, *p* < 0.01, 95% CI [−0.173, −0.038]) and higher feelings of self-worth (*b* = 0.472, *p* < 0.001, 95% CI [0.283, 0.661]). A significant interaction effect was observed in the self-worth and EIC model (*b* = −0.013, *p* < 0.01, 95% CI [−0.023, −0.003]), with results indicating that EIC exacerbated the association between same-race rejection and feelings of self-worth. Figure 1 displays the conditional effects of same-race rejection on self-worth at low, moderate, and high levels of EIC. Participants who reported high levels of EIC (*b* = −0.015, *p* < 0.01, 95% CI [−0.025, −0.004]) scored significantly lower on feelings of self-worth. Moderate and low levels of EIC did not significantly modify this association. All four same-race rejection models were significant (*p* < 0.001) and explained 26–37% of the variance in depressive symptoms and self-worth (see Table 3).

#### 3.2.3. White Physical Objectification

**Figure 2:**
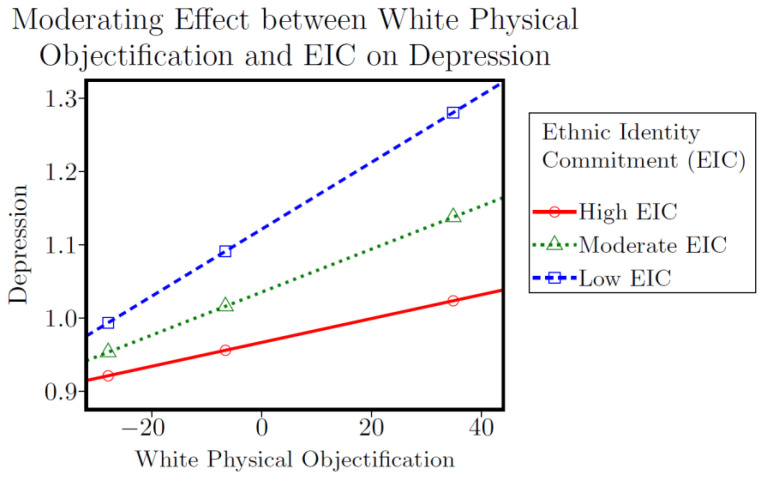
Effect of White Physical Objectification X EIC on Depression.

Higher scores on White physical objectification were associated with higher depressive symptoms in both the EIS (*b* = 0.003, *p* < 0.001, 95% CI [0.001, 0.005]) and EIC models (*b* = 0.003, *p* < 0.001, 95% CI [0.002, 0.005]). Higher scores on White physical objectification were also associated with lower feelings of self-worth in both the EIS (*b* = −0.005, *p* < 0.05, 95% CI [−0.009, −0.000]) and EIC models (*b* = −0.006, *p* < 0.05, 95% CI [−0.010, −0.001]). Higher scores on EIC were associated with lower depressive symptoms (*b* = −0.120, *p* < 0.001, 95% CI [−0.189, −0.052]) and higher feelings of self-worth (*b* = 0.493, *p* < 0.001, 95% CI [0.301, 0.685]). A significant interaction effect was observed in the depression and EIC model (*b* = −0.002, *p* < 0.05, 95% CI [−0.005, 0.000]), with results indicating that EIC attenuated the association between White physical objectification and depressive symptoms. Figure 2 displays the conditional effects of White physical objectification on depressive symptoms at low, moderate, and high levels of EIC. Participants who reported moderate (*b* = 0.003, *p* < 0.001, 95% CI [0.001, 0.004]) and high (*b* = 0.005, *p* < 0.001, 95% CI [0.002, 0.007]) levels of EIC scored significantly lower on depressive symptoms. Low levels of EIC did not significantly modify this association. All four White physical objectification models were significant (*p* < 0.001) and explained 26–36% of the variance in depressive symptoms and self-worth (see Table 4). 

#### 3.2.4. Covariates

Age, perceived rejection, and self-perceived sexual attractiveness were significant across all twelve regression models. No other covariates emerged as significant. Across all models, age was associated with lower depressive symptoms (*p* < 0.001) and higher feelings of self-worth (*p* < 0.01); perceived rejection was associated with higher depressive symptoms (*p* < 0.001) and lower feelings of self-worth (*p* < 0.001); and self-perceived sexual attractiveness was associated with lower depressive symptoms (*p* < 0.001) and higher feelings of self-worth (*p* < 0.001).

## 4. Discussion

This study aimed to examine ethnic identity as a moderator of the association between RSD and psychological well-being among a large sample of YSMBM. Researchers have provided substantial evidence for the protective qualities of ethnic identity, but some researchers have also reported that certain aspects of ethnic identity may exacerbate the effects of certain racialized stressors [39,40,43,58,59]. We estimated twelve hierarchical linear regression models in total, using three RSD subscales, two psychological well-being outcomes, and two subscales of ethnic identity. We hypothesized that ethnic identity commitment would attenuate the association between RSD and well-being, while ethnic identity exploration would exacerbate the association between RSD and well-being.

### 4.1. Main Effects

All study covariates performed identically to previous main effects analyses examining the relationship between RSD and psychological well-being. We discuss the implications of these associations at length in prior published work [31]. In addition, all three RSD subscales were associated with poorer psychological well-being (**H1**), consistent with our prior work, as well as other scholars’ work examining sexual racism among sexual minority men of color [24,25,26,27,28,29].

Participants reported overall moderate to high scores on both ethnic identity subscales. This coincides with extensive research showing that Black sexual minority individuals tend to identify strongly with their racial/ethnic identity [56,74]. The identity commitment subscale was significantly associated with higher feelings of self-worth and lower depressive symptoms in all twelve models. The identity search subscale, however, was significantly associated with higher feelings of self-worth, but only in the White physical objectification model; it failed to achieve significance in the other eleven models. These findings are consistent with prior research indicating that the sense of belonging, pride, and commitment towards one’s ethnic background appears to be health promotive for Black Americans [47,49,51]. By comparison, the fewer significant findings for identity search suggest that this particular aspect of ethnic identity is not as strongly associated with positive psychological well-being, which has been reported in prior research [39,46]. While other researchers have indicated that identity search may, in fact, be associated with less psychological distress, many of these studies have only investigated identity search in a composite measure combined with other aspects of identity, and/or have only noted these associations among participants who do not identify as Black [45,75,76,77,78,79]. For YSMBM, our findings indicate that ethnic identity search may have limited bearing on depressive symptoms or feelings of self-worth in the context of RSD.

### 4.2. Moderation Effects

Contrary to our hypothesis (**H2**), higher scores on identity search did not significantly exacerbate the association between RSD and psychological well-being in any model. Although our findings do not support the prior theoretical and empirical work in which we grounded our hypothesis [39,40,43,80], it is nevertheless encouraging that a factor thought to exacerbate racial stress may be inconsequential among YSMBM—at least with respect to the health outcomes under investigation. As previously discussed, ethnic identity search is not inherently negative, though the rationale behind predicting an exacerbation effect is well situated when *uncertainty* is thought to be the underlying component of exploration. Scholars have noted, however, that certain aspects of identity that have a more definitive positive valence (e.g., identity commitment), are not only highly correlated with identity search, but may have a reciprocal relationship with identity search [81]. For example, having a firm commitment to one’s ethnic identity may encourage individuals to explore more about their ethnic group heritage; conversely, through the process of exploring one’s background, individuals may arrive at a stronger sense of commitment. These two closely related aspects of identity are part of the reason why the MEIM is so often used as a composite measure in ethnic identity research. When disaggregated, however, researchers are able to parse out the effects of these two related but distinct constructs. In the process, it has come to light that identity search may not necessarily have the same protective qualities as identity commitment, and may in fact exacerbate a stressor—or, in our case, exert no influence at all. Altogether, our findings offer additional clarity on how ethnic identity search operates in the context of RSD as experienced by YSMBM.

In contrast to our null findings for identity search, our results do suggest that identity commitment may significantly modify the relationship between RSD and psychological well-being. Moderate to high levels of commitment buffered the association between White physical objectification and depressive symptoms among the study sample, thus partially supporting one of our central hypothesis (**H3**). This is consistent with prior research illustrating that ethnic identity may serve as a potential safeguard against racialized stress [38,39,40,46,51,54,55], and may therefore be of interest to mental health scholars/practitioners who work with YSMBM. Being physically objectified by White men is not only one of the most dominant themes in the broader discourse on sexual racism [14,15,82,83,84], but this subscale also had the largest effect size of any RSD subscale in our original main effects analysis [31]. Thus, identifying and strengthening factors that may mitigate the adverse effects of White physical objectification may be a priority area for sexual racism researchers and interventionists.

Although the buffering hypothesis received partial support, our moderation results did reveal an unexpected finding: identity commitment exacerbated the association between same-race rejection and lower feelings of self-worth, rendering this interaction significant for participants who reported high levels of commitment. Though contrary to our hypothesis, this does coincide with some literature indicating that certain aspects of ethnic identity (e.g., centrality of identity, affective identity pride) may exacerbate the effects of racial discrimination [85,86,87]. Moreover, prior scholarship has predominantly focused on the protective qualities of ethnic identity commitment using measures of discrimination that do not distinguish between in-group and out-group discrimination [55,88,89,90]. The role of ethnic identity in response to in-group discrimination is likely more complex, and, to our knowledge, has not been investigated quantitatively among YSMBM, nor within the context of intimate partner seeking in general. In our earlier qualitative work, YSMBM expressed complicated feelings with respect to being rejected by members of their same race or from other racial/ethnic minority men, ranging from bewilderment to despondency and disbelief [65]. It is possible that YSMBM who experience a strong sense of ethnic identity may put greater stock into being accepted by other Black men, or generally expect that Black men would be more likely to desire them as a partner; thus, they may be more negatively impacted by experiencing rejection from them. In contrast, YSMBM with a lower sense of commitment to their ethnic identity may place less value on being selected as a mate by other Black men, and are therefore less significantly impacted if/when they experience rejection from them. Ultimately, a key takeaway from these findings is that certain aspects of identity—even those that are conceptualized as inherently positive—may not behave in a uniform manner in the presence of discrimination. The ways in which identity serves to buffer or exacerbate the effects of identity-related stress may be contingent upon the specific nature of the stressor, as well the source of that stress.

### 4.3. Implications

These findings contribute to a growing body of research into the associations between ethnic identity and well-being among sexual minority people of color [56,91], and may have important implications for individual and group-level interventions. It may be critical for clinicians who work with YSMBM to engage, affirm, and leverage ethnic belonging in facing objectification and other forms of discrimination from White men. Clinical approaches based on cognitive behavioral therapy (CBT) may be particularly helpful in enabling YSMBM to leverage their sense of ethnic belonging when coping with RSD. Many researchers have adapted CBT for Black clients and for sexual minority clients, and it has proven effective in helping clients cope with discrimination [92,93,94,95,96]. However, research on CBT as an intervention for discrimination among sexual minority people of color is in its infancy. In a recent pilot test, Jackson et al. tested a CBT-informed group therapy intervention for coping with intersectional stigmas among Black and Latino sexual minority men [93]. Most participants reported improvements in mental health symptoms—including anxiety, depression, and suicidality—and many reported decreased identity-related stress. Such interventions may be applied in clinical settings to address experiences of RSD, and may also integrate ethnic identity as a component of the treatment process.

YSMBM with high ethnic identity commitment may require support in grappling with same-race rejection while maintaining the overall benefits of ethnic identity commitment. Critical consciousness interventions, which support participants in understanding and opposing oppressive forces, may help them to understand interpersonal discrimination in terms of broader structures of White supremacy. Various researchers have advocated for critical consciousness interventions in addressing racism and other forms of oppression [97,98]. In a recent study, Goodkind et al. evaluated a critical consciousness program for Black girls in high school. The program was designed to enhance participants’ well-being, as well as their capacity to engage in critical reflection related to race- and gender-related stress [99]. The researchers found that participants were more likely to reframe racist encounters as an injustice rather than internalizing these experiences, and participants were also motivated to organize amongst themselves to address racially mediated mistreatment. Critical consciousness programs such as these could address RSD through analysis of the objectification of Black men in popular media, or exploration of same-race discrimination as a facet of White supremacy throughout American history. Successful critical consciousness interventions focused on RSD may, in turn, enhance YSMBM’s self-efficacy to confront, resist, or subvert patterns of discrimination perpetuated in online settings through individual or collective action.

Critical consciousness interventions are not limited to those who are subject to oppression, but may also be fostered among health professionals and clinical practitioners. A robust cultivation of critical consciousness on the part of clinicians may enable them to more effectively engage with YSMBM, and help them navigate complex intersectional stressors that they encounter in intimate partner-seeking contexts [100]. Moreover, cultural competency and cultural humility training remains imperative for practitioners, particularly those who do not hold sexual or racial/ethnic minority identities [101,102]. Altogether, effective clinical interventions that engage with RSD and ethnic identity call for a high degree of critical awareness, sensitivity, and reflexivity on the part of providers.

## 5. Strengths and Limitations

To our knowledge, this is the first study to examine ethnic identity as a moderator of the association between RSD and psychological well-being among YSMBM. It is also among the first to examine the association between ethnic identity and feelings of self-worth and depressive symptoms in any sexual minority population. Our disaggregation of the search and commitment subscales of the MEIM adds further clarity to the ethnic identity literature, as the majority of studies using the MEIM use the scale as a composite measure [40]. The study’s large national sample and the breadth of the RSD scale in measuring various forms of discrimination were also significant strengths. Notably, the RSD scale used in this study distinguishes between expressions of discrimination to a greater degree than most measures of discrimination. This distinction between race of perpetrator is rare in research on RSD, and thus constitutes a unique contribution to the field.

This study, however, is not without its limitations. The study’s cross-sectional design and lack of representative sample limits generalizability, as well as our capacity to make causal inferences about the relationships between ethnic identity, RSD, and well-being among YSMBM. The study is further limited by its all-Black sample and by its focus on RSD as perpetrated by Black or White users online; thus, the findings cannot generalize to other racial/ethnic groups, nor can they provide insights into RSD as perpetuated by racial/ethnic groups other than Black or White men. The MEIM is also not without critique. The scale has been subject to revisions, including both expanding and shortening the 12-item version used in this study, and researchers have also reported slightly different factor solutions for the scale among different populations [81,103]. Moreover, competing theoretical frameworks of ethnic identity (e.g., social identity theory, self-categorization theory) and their corresponding measures could produce different results than reported here. To arrive a more robust understanding of ethnic identity and its role in the context of RSD, it will be important to consider different identity measures and apply different theoretical frameworks in future work.

## 6. Directions for Future Research

Although the health and social science literature on RSD has been largely qualitative, there are still many important questions about RSD that call for qualitative or mixed methodological approaches. Most qualitative research on RSD among sexual minority men has focused on the experience of discrimination, but only a handful of studies have focused on strengths for coping with RSD, including identity-based strengths [11,13,22]. Carefully designed qualitative studies will enable researchers to ascertain the types of strategies that sexual minority men of color are leveraging to mitigate the adverse effects of RSD, as well as identify other intrapersonal characteristics that may be protective in the context of RSD. Such studies may add important nuance to existing quantitative findings, inform future quantitative studies, and provide a foundation for intervention research and clinical application.

A closer examination of same-race rejection may be especially important for sexual racism researchers, particularly in light of our unexpected findings. Although commitment to one’s ethnic identity exacerbates the effect of same-race rejection, its overall effect is positive, and YSMBM struggling in the face of same-race rejection are not likely to benefit from lessening their commitment. Given the complexity inherent in navigating this experience, it may be more beneficial to focus on the motivations of YSMBM who are inclined to reject members of their same race. As with other forms of internalized racism, perpetrators of same-race rejection are devalued by the same system of White supremacy in which they participate. However, only a small amount of research on RSD examines same-race discrimination [104,105,106]. Research on both in-group and out-group perpetrators of sexual racism has usually focused on refuting their actions or examining their pathology rather than on interventions that might change their actions and ideologies [5,13,107,108,109]. However, intervention research aiming to address general internalized racism has shown promise. In a recent pilot study, Banks et al. examined an acceptance commitment therapy (ACT) intervention designed to address internalized racism among Black women. They found decreases in internalized oppression, internalized shame, and negative psychological symptoms [110]. Similar interventions may be applicable to YSMBM, and researchers may wish to examine the degree to which such interventions reduce instances of same-race rejection in intimate partner-seeking contexts.

Future research should also examine ethnic identity, RSD, and health among other sexual minority men of color. Patterns of RSD may manifest differentially across different racial/ethnic groups (e.g., certain stereotypes and sexual scripts are ascribed to certain groups and not others), and some groups may respond differently to RSD than others. For some racial/ethnic minority groups, generational status—or recency of immigration—should also be examined in the context of RSD. Generational status may have some bearing on both self-reported ethnic identification and exposure to RSD over time [85,111,112]. Moreover, there is a large and complex literature base examining how acculturation and acculturative stress relate to experiences of discrimination and psychosocial functioning among Asian and Latino populations, and these processes may be distinct from the experiences of Black Americans [113,114,115,116]. Studies should also be conducted in other regional contexts, as racial hierarchies in other parts of the world are distinct from those in the United States [20,107].

Using intersectional identity measures (e.g., a measure of both racial/ethnic and sexual orientation identity in tandem) will also add important nuance to work of this nature moving forward. There is a marked deficit of intersectional measures for sexual and gender minority populations, but scholars have long highlighted the importance of examining overlapping identities instead of focusing on singular markers of identity [56,117,118,119]. An intersectional analysis will enable researchers to quantitatively model how specific intersecting identity statuses operate in the context of RSD—a key distinction when examining sexual minority men of color across multiple racial/ethnic groups. It will also be important to examine factors other than identity that may exert influence over the relationship between RSD and psychological well-being. Social support, for example, is widely regarded as a critical protective asset in the health literature [120,121,122]. However, there is limited research that explores how sexual minority men of color respond to instances of RSD, and/or if they seek out support networks to help process these experiences [4,6,25,123,124]. Understanding how social support operates in the context of RSD—both in terms of the presence and/or absence of support networks, as well as the active leveraging of support—may provide important avenues for intervention.

## 7. Conclusions

Overall, this study provides valuable insight into the relationship between ethnic identity, RSD, and psychological well-being. Moving forward, researchers should continue to account for factors that may further elucidate these relationships. They should also explore multi-pronged strategies to disrupt harmful behavior perpetrated online. RSD, like any form of racialist discrimination, is neither natural nor inevitable, and combatting racialist ideologies at their root remains critical to advance health equity for marginalized populations.

## Figures and Tables

**Table 1:** Descriptive Statistics for Study Sample.

Categorical Variables	*n* (*M*)	% (*SD*)			
Sexual Orientation					
Gay	491	71.2%			
Bisexual	111	16.1%			
Other	90	12.7%			
Education					
Less than high school	1	0.1%			
High school graduate	78	11.3%			
Some college	294	42.6%			
College graduate	209	30.3%			
Post college	108	15.7%			
App Use					
Once a month or less	183	26.5%			
2–3 times a month	132	19.1%			
About once a week	74	10.7%			
2–6 times a week	121	17.5%			
About once a day	64	9.3%			
More than once a day	116	16.8%			
Relationship Status (single)	593	85.9%			
HIV Status (positive)	100	14.5%			
**Continuous Variables**	***M***	***SD***	**Min**	**Max**	**α**
Age	24.47	3.18	18	29	—
Perceived Rejection	1.49	0.81	0	4	0.761
Perceived Attractiveness	5.06	1.62	1	7	0.951
Moderator Variables					
EI Commitment	3.28	0.63	1	4	0.907
EI Search	3.08	0.64	1	4	0.780
Dependent Variables					
Depressive Symptoms	1.05	0.63	0	2.90	0.920
Feelings of Self-Worth	5.56	1.87	0	8	0.950
RSD Subscales					
White Superiority	32.03	18.42	0	87.5	0.833
Same-Race Rejection	26.55	16.71	0	100	0.851
White Physical Obj.	39.13	27.69	0	100	0.857

**Table 2:** White Superiority and Ethnic Identity on Depression and Self-Worth.

	Depression & EIS				Self-Worth & EIS				Depression & EIC				Self-Worth & EIC			
	*b*	*SE*	LL	UL	*b*	*SE*	LL	UL	*b*	*SE*	LL	UL	*b*	*SE*	LL	UL
(Constant)	1.677 ***	0.238	1.210	2.144	3.270 ***	0.673	1.949	4.591	1.672 ***	0.236	1.208	2.136	3.267 ***	0.663	1.966	4.568
**Covariates**																
Age	−0.025 ***	0.008	−0.010	−0.011	0.059 **	0.021	0.017	0.101	−0.026 ***	0.007	−0.040	−0.011	0.059 **	0.021	0.018	0.099
Education	−0.023	0.027	−0.075	0.029	0.003	0.075	−0.144	0.150	−0.019	0.026	−0.070	0.032	−0.002	0.073	−0.146	0.141
HIV+	0.015	0.060	−0.103	0.133	−0.198	0.170	−0.532	0.136	−0.009	0.060	−0.127	0.109	−0.108	0.169	−0.440	0.223
App Use	0.016	0.012	−0.006	0.039	−0.052	0.033	−0.117	0.012	0.015	0.012	−0.008	0.037	−0.045	0.032	−0.109	0.018
Single	−0.054	0.061	−0.174	0.065	0.068	0.171	−0.268	0.405	−0.047	0.060	−0.165	0.072	0.049	0.170	−0.284	0.382
PR	0.256 ***	0.030	0.198	0.315	−0.576 ***	0.085	−0.741	−0.410	0.253 ***	0.030	0.194	0.311	−0.555 ***	0.083	−0.719	−0.391
SPSA	−0.070 ***	0.015	−0.100	−0.041	0.436 ***	0.042	0.353	0.519	−0.062 ***	0.015	−0.091	−0.032	0.404 ***	0.042	0.321	0.486
**Predictors**																
WS	0.003 **	0.002	0.001	0.006	−0.004	0.004	−0.011	0.003	0.004 **	0.001	0.001	0.006	−0.004	0.003	−0.011	0.002
EIS	−0.030	0.034	−0.098	0.037	0.182	0.097	−0.009	0.373								
EIC									−0.104 **	0.035	−0.172	−0.037	0.462 ***	0.097	0.272	0.652
**Interactions**																
WS × EIS	−0.003	0.002	−0.006	0.000	0.005	0.005	−0.005	0.015								
WS × EIC									−0.002	0.002	−0.005	−0.001	0.002	0.005	−0.007	0.010
Model fit	*R*^2^ = 0.263, MSE = 0.295 *F* (10,679) = 24.225 ***				*R*^2^ = 0.337, MSE = 2.361 *F* (10,679) = 34.589 ***				*R*^2^ = 0.270, MSE = 0.292 *F* (10,679) = 25.137 ***				*R*^2^ = 0.355, MSE = 2.298 *F* (10,679) = 37.384 ***			
	*R*^2^ change = 0.0033 *F* (1679) = 3.074				*R*^2^ change = 0.0011 *F* (1679) = 1.102				*R*^2^ change = 0.0015 *F* (1679) = 1.442				*R*^2^ change = 0.0001 *F* (1679) = 0.112			

Notes. *b* = unstandardized regression coefficients, *SE* = standard error, LL = low limit, UL = upper limit, PR = Perceived Rejection, SPSA = Self-Perceived Sexual Attractiveness, WS = White Superiority, EIS = Ethnic Identity Search, EIC = Ethnic Identity Commitment. Referent groups are being in a relationship (Single); negative HIV status (HIV+). ** *p* < 0.01; *** *p* < 0.001.

**Table 3:** Same-Race Rejection and Ethnic Identity on Depression and Self-Worth.

	Depression & EIS				Self-Worth & EIS				Depression & EIC				Self-Worth & EIC			
	*b*	*SE*	LL	UL	*b*	*SE*	LL	UL	*b*	*SE*	LL	UL	*b*	*SE*	LL	UL
(Constant)	1.604 ***	0.237	1.138	2.071	3.345 ***	0.675	2.020	4.670	1.599 ***	0.235	1.137	2.061	3.420 ***	0.661	2.122	4.719
**Covariates**																
Age	−0.026 ***	0.008	−0.040	−0.011	0.059 **	0.021	0.018	0.101	−0.025 ***	0.007	−0.040	−0.010	0.054 **	0.021	0.013	0.095
Education	−0.013	0.026	−0.065	0.038	−0.008	0.075	−0.155	0.138	−0.014	0.026	−0.064	0.037	0.005	0.073	−0.138	0.147
HIV+	0.037	0.060	−0.081	0.155	−0.216	0.170	−0.551	0.118	0.016	0.060	−0.101	0.134	−0.135	0.168	−0.465	0.195
App Use	0.017	0.012	0.005	0.040	−0.055	0.033	−0.120	0.009	0.015	0.011	−0.008	0.037	−0.046	0.032	−0.110	0.017
Single	−0.039	0.061	−0.158	0.080	0.062	0.173	−0.277	0.400	−0.037	0.060	−0.155	0.082	0.058	0.169	−0.274	0.390
PR	0.245 ***	0.030	0.185	0.304	−0.575 ***	0.086	−0.743	−0.406	0.240 ***	0.030	0.181	0.299	−0.557 ***	0.084	−0.722	−0.392
SPSA	−0.070 ***	0.015	−0.099	−0.042	0.439 ***	0.042	0.357	0.521	−0.062 ***	0.015	−0.091	−0.033	0.402 ***	0.042	0.320	0.484
**Predictors**																
SRR	0.005 ***	0.001	0.003	0.008	−0.004	0.004	−0.011	0.004	0.006 ***	0.001	0.003	0.008	−0.005	0.004	−0.013	0.002
EIS	−0.021	0.034	−0.087	0.044	0.159	0.095	−0.028	0.346								
EIC									−0.106 **	0.034	−0.173	−0.038	0.472 ***	0.096	0.283	0.661
**Interactions**																
SRR × EIS	0.001	0.002	−0.003	−0.004	−0.000	0.005	−0.011	−0.010								
SRR × EIC									0.003	0.002	−0.001	0.006	−0.013 **	0.005	−0.023	−0.003
Model fit	*R*^2^ = 0.269, MSE = 0.293 *F* (10,679) = 24.995 ***				*R*^2^ = 0.336, MSE = 2.365 *F* (10,679) = 34.401 ***				*R*^2^ = 0.280, MSE = 0.288 *F* (10,679) = 26.458 ***				*R*^2^ = 0.361, MSE = 2.276 *F* (10,679) = 38.424 ***			
	*R*^2^ change = 0.0001 *F* (1679) = 0.132				*R*^2^ change = 0.0000 *F* (1679) = 0.004				*R*^2^ change = 0.0024 *F* (1679) = 2.310				*R*^2^ change = 0.0067 *F* (1679) = 7.093 **			

Notes. *b* = unstandardized regression coefficients, *SE* = standard error, LL = low limit, UL = upper limit, PR = Perceived Rejection, SPSA = Self-Perceived Sexual Attractiveness, SRR = Same-Race Rejection, EIS = Ethnic Identity Search, EIC = Ethnic Identity Commitment. Referent groups are being in a relationship (Single); negative HIV status (HIV+). ** *p* < 0.01; *** *p* < 0.001.

**Table 4:** White Physical Objectification and Ethnic Identity on Depression and Self-Worth.

	Depression & EIS				Self-Worth & EIS				Depression & EIC				Self-Worth & EIC			
	*b*	*SE*	LL	UL	*b*	*SE*	LL	UL	*b*	*SE*	LL	UL	*b*	*SE*	LL	UL
(Constant)	1.720 ***	0.238	1.253	2.187	3.225 ***	0.674	1.902	4.548	1.734 ***	0.236	1.271	2.197	3.210 ***	0.663	1.908	4.511
**Covariates**																
Age	−0.027 ***	0.008	−0.041	−0.012	0.060 **	0.021	0.018	0.102	−0.026 ***	0.007	−0.041	−0.012	0.058 **	0.021	0.018	0.099
Education	−0.023	0.026	−0.074	0.029	0.002	0.075	−0.144	0.148	−0.023	0.026	−0.074	0.028	0.003	0.073	−0.140	0.146
HIV+	0.026	0.060	−0.091	0.144	−0.210	0.170	−0.543	0.123	0.001	0.060	−0.116	0.119	−0.120	0.168	−0.450	0.210
App Use	0.015	0.012	−0.007	0.038	−0.049	0.033	−0.114	0.015	00.13	0.012	−0.010	0.035	−0.040	0.032	−0.104	0.023
Single	−0.074	0.060	−0.193	0.044	0.091	0.171	−0.250	0.427	−0.066	0.060	−0.183	0.052	0.070	0.168	−0.261	0.400
PR	0.262 ***	0.029	0.205	0.320	−0.576 ***	0.083	−0.739	−0.412	0.255 ***	0.029	0.197	0.312	−0.547 ***	0.032	−0.708	−0.385
SPSA	−0.079 ***	0.015	−0.103	−0.045	0.439 ***	0.042	0.358	0.521	−0.065 ***	0.015	−0.095	−0.036	0.406 ***	0.042	0.325	0.488
**Predictors**																
WO	0.003 ***	0.001	0.001	0.005	−0.005 *	0.002	−0.009	−0.000	0.003 ***	0.001	0.002	0.005	−0.006 *	0.002	−0.010	−0.001
EIS	−0.037	0.034	−0.105	0.030	0.198 *	0.097	0.007	0.388								
EIC									−0.120 ***	0.035	−0.189	−0.052	0.493 ***	0.098	0.301	0.685
**Interactions**																
WO × EIS	−0.002	0.001	−0.005	0.000	0.002	0.003	−0.005	0.008								
WO × EIC									−0.002 *	0.001	−0.005	0.000	0.000	0.003	−0.006	0.007
Model fit	*R*^2^ = 0.269, MSE = 0.293 *F* (10,679) = 24.929 ***				*R*^2^ = 0.340, MSE = 2.354 *F* (10,679) = 34.916 ***				*R*^2^ = 0.280, MSE = 0.289 *F* (10,679) = 26.379 ***				*R*^2^ = 0.360, MSE = 2.282 *F* (10,679) = 38.132 ***			
	*R*^2^ change = 0.0041 *F* (1679) = 3.801				*R*^2^ change = 0.0003 *F* (1679) = 0.298				*R*^2^ change = 0.0042 *F* (1679) = 4.006 *				*R*^2^ change = 0.0000 *F* (1679) = 0.003			

Notes. *b* = unstandardized regression coefficients, *SE* = standard error, LL = low limit, UL = upper limit, PR = Perceived Rejection, SPSA = Self-Perceived Sexual Attractiveness, WO = White Objectification, EIS = Ethnic Identity Search, EIC = Ethnic Identity Commitment. Referent groups are being in a relationship (Single); negative HIV status (HIV+). * *p* < 0.05; ** *p* < 0.01; *** *p* < 0.001.

## Data Availability

The data presented in this study are available on request from the corresponding author.
